# The impact of United States Medical Licensing Exam (USMLE) step 1 cutoff scores on recruitment of underrepresented minorities in medicine: A retrospective cross‐sectional study

**DOI:** 10.1002/hsr2.161

**Published:** 2020-04-20

**Authors:** Myia Williams, Eun Ji Kim, Karalyn Pappas, Omolara Uwemedimo, Lyndonna Marrast, Renee Pekmezaris, Johanna Martinez

**Affiliations:** ^1^ Department of Medicine Northwell Health Manhasset New York; ^2^ Zucker School of Medicine at Hofstra/Northwell Hempstead New York; ^3^ The Feinstein Institutes for Medical Research Manhasset New York

**Keywords:** disparity, diversity, race/ethnicity, URiM, USMLE

## Abstract

**Background and Aims:**

United States Medical Licensing Exam (USMLE) scores are the single, most objective criteria for admission into residency programs in the country. Underrepresented minorities in medicine (URiM) are found to have lower USMLE scores compared to their White counterparts. The objective of this study is to examine how USMLE step 1 cutoff scores may exclude self‐reported URiM from the residency interview process across various specialties.

**Methods:**

This was a retrospective cross‐sectional study of 10 541 applicants to different residency programs at Zucker School of Medicine at Hofstra/Northwell Health between May 2014 and May 2015. We identified Blacks and Hispanics as URiM. The primary outcome is the percentage of applicants with USMLE step 1 score above different ranges of cutoff score, from 205 to 235 in five‐point increments, by race/ethnicity and by URiM status. Secondary outcome is percentages of URiM vs non‐URiM above and below mean USMLE step 1 scores by different specialties (internal medicine, obstetrics/gynecology, pediatrics, and psychiatry).

**Results:**

The study sample included 2707 White, 722 Black, 805 Hispanic, 5006 Asian, and 562 Other Race/Ethnicity applicants. Overall, 50.2% were male, 21.3% URiM, 7.4% had limited English proficiency, 67.6% attended international medical schools, and 2.4% are Alpha Omega Alpha Honor Medical Society (AOA) members. The mean (±SD) USMLE step 1 score was significantly greater among non‐URiM applicants as compared to URiM applicants (223.7 ± 19.4 vs 216.1 ± 18.4, *P* < .01, two‐sample *t*‐test). Non‐URiM applicants were younger, and the percentage of male and AOA applicants was greater among non‐URiM applicants as compared to URiM applicants (50.5% vs 47.7%, *P* = .02, Chi‐Square test; 2.9% vs 1.2%, *P* < .01, Chi‐Square test, respectively).

**Conclusion:**

Using a USMLE step 1 cutoff score as an initial filter for applicant recruitment and selection could jeopardize the benefits of a diverse residency program. Practical implications are discussed.

## 1 INTRODUCTION

Research has consistently shown that a large segment of ethnic and racial minorities in the United States (US) population face inequities in both health care quality and access.^1^, ^2^, ^3^ A growing body of empirical evidence has shown that increasing the diversity of the physician workforce may assist in eliminating health disparities in the US.^1^, ^2^, ^4^, ^5^, ^6^, ^7^ For example, results from previous research indicates that minority physicians are more likely to care for minority patients and work in underserved communities.^4^, ^7^ Patient‐provider concordance can produce better communication, trust, satisfaction, adherence to medication, and health outcomes, supporting the case for diversity.^1^, ^8^, ^9^, ^10^ Despite the many benefits of a diverse physician workforce, there are various factors that limit it.^11^, ^12^, ^13^ At the Graduate Medical Education (GME) level, one potential limiting factor of a diverse physician workforce is the overemphasis on standardized test scores such as the United States Medical and Licensing Examination (USMLE).^1^, ^11^, ^14^, ^15^, ^16^

Administered by the National Board of Medical Examiners (NBME), the USMLE is a series of three tests (steps 1‐3) for the purpose of granting medical licenses to physicians who want to practice in the US.^11^, ^16^, ^17^ The USMLE step 1 is intended to assess medical students' understanding and application of basic science concepts to medical practice, with a special emphasis on principles underlying modes of disease, therapy, and health.^15^, ^16^, ^17^ All residency program applicants graduating from allopathic schools are required to take USMLE step 1, with many program directors using USMLE and the Comprehensive Osteopathic Medical Licensing Examination (COMLEX‐USA; for osteopathic school) scores in making their decisions to grant interviews.^11^, ^15^, ^18^, ^19^ The NBME recognizes the use of step 1 scores as “a major factor in residency screening and selection,” which may be useful to some key stakeholders but viewed as a negative consequence for others, such as those underrepresented minorities in medicine (URiM).^15^, ^20^

The Association of American Medical Colleges (AAMC) defines URiM as “racial and ethnic populations that are underrepresented in the medical profession relative to their numbers in the general population.”^21^ URiM include individuals from African American (AA), Hispanic/Latino (HL), and Native American racial and ethnic groups.^21^ According to the 2016 U.S. Census data, racial and ethnic minorities comprised at least 38.7% of the U.S. population, 17.8% of which were H/L, 13.3% Black/AA, and 2% Native Americans^22^, ^23^ Yet, between 1997 and 2017, AA, H/L, and Native Americans made up only 4%, 4%, and <0.04% of medical doctors, respectively.^1^, ^2^, ^24^ In 2014, the population of AA and H/L in New York City, one of the most ethnically diverse cities in the US, was 53%, yet, only 12% of practicing physicians were URiM.^2^, ^24^

Since the implementation of step 1 in the 1990s, exam results were reported as a pass/fail status, but with time, it also included a three‐digit numeric score.^11^, ^14^ Typically, URiM students score lower on standardized tests than do White students.^11^, ^25^ One study found that based on USMLE cutoff scores, AA were three to six times less likely to be offered an interview compared to non‐AA; White students had a mean score of 210, while Black students had a mean score of 187.9.^11^ A 2019 study by the NBME showed that compared to White males, female students scored 5.9 points lower, while Asians, H/L, and Black test‐takers scored 4.5, 12.1, and 16.6 points lower, respectively, on the USMLE step 1.^26^

In this study, we explore how using cutoffs to screen applicants may affect the number of URiM participating in the interview process for residency programs at a single institution. We focus on the potential bias in USMLE step 1 cutoff scores across racial/ethnic groups and medical specialties. Despite the importance of racial/ethnic biases in the USMLE, only one study has previously examined the racial/ethnic biases of USMLE step 1 scores.^11^ The study was limited by using socially assigned race by examining photographs of the applicants to determine whether they were African American or non‐African American.^11^ The standard in the US is for one to self‐report their racial/ethnic identity. We built on the previous study by: (a) using the Electronic Residency Application Service (ERAS)^27^ self‐reported racial category to classify participants into URiM vs non‐URiM groups, (b) increasing statistical power with a larger sample size, and (c) examining if biases in cutoff scores exist across specialties. This study is important to shed light on one of the most important factors in the selection process for residency program applications in the US, which will eventually help pave the way for more opportunities in serving disparity populations within the medical field.

## 2 METHODS

In this retrospective cross‐sectional study, we extracted data from ERAS of 10 541 residency applicants applying to five residency programs at Zucker School of Medicine at Hofstra/Northwell Health between May 2014 and May 2015. ERAS is the centralized online application service that medical students around the world use to deliver their application, along with supporting documents, to residency programs in the US.^27^ This study was conducted as part a larger institution‐wide diversity and equity strategy. The programs were selected because they represent the largest programs for our academic institution. Residency programs studied included internal medicine (two sites), pediatrics, obstetrics‐gynecology, and psychiatry residencies at North Shore University Hospital, Long Island Jewish Medical Center, and Forest Hills Hospital. Northwell Health is located in the New York metropolitan area and is the third largest health care system nationally. The program has more than 1800 residents and fellows, serving patients at 23 hospitals.

The primary outcome was USMLE step 1 score. For applicants with more than one USMLE step 1 score, we used their highest USMLE step 1 score. Self‐reported race/ethnicity was categorized into five groups: non‐Hispanic White, non‐Hispanic Black, Hispanic, Asian, and Other. This categorization was based off of the ERAS predetermined variables, which are “self‐identified” by applicants. Using the AAMC definition,^21^ URiM were defined as those in the Non‐Hispanic Black, Hispanic, and Other categories. Non‐URiM were defined as those in Asian or White category.

Based on previous research,^11^ we also identified key sociodemographic characteristics associated with USMLE step 1 scores. They include age, sex/gender, limited English proficiency status, international medical school attendance, and Alpha Omega Alpha (AOA) status. We calculated age as of 1 September 2014 using applicants' date of birth. Sex was categorized as male, female, or missing; while ERAS technically records “gender,” for the purposes of this study, since we are reporting male/female, we used the term “sex.” English proficiency status was defined as English native/functionally native, or not English native/functionally native; international medical school attendance was categorized as trained in the US (any medical school training in the US) or not trained in the US (no medical school training in the US).

We performed descriptive analyses (means and standard deviations or medians and interquartile ranges) of residency applicants and then subgroup analyses by race/ethnicity category and URiM status. USMLE step 1 scores are normally distributed, therefore, one‐way analysis of variance (ANOVA) was used to compare the mean USMLE step 1 scores for each race/ethnicity, and two‐sample *t*‐test was used to compare mean USMLE step 1 score by URiM status. Next, we examined USMLE step 1 distributions by medical specialties. To examine how USMLE step 1 cutoff scores would affect the number of applicants qualifying for a potential interview, we calculated the percentage of applicants with USMLE step 1 score above different ranges of cutoff score: 205, 210, 215, 220, 225, and 230; these were conveniently determined for ease of analysis.

Lastly, we calculated mean USMLE step 1 score for different medical specialties and compared percentage of applicants above the mean USMLE step 1 score by URiM status. The Chi‐Square test was used to compare percentages by groups. The ANOVA test was used to compare the means of continuous variables by group. For non‐normally distributed data, the nonparametric Wilcoxon Signed Rank Sum test or Kruskal‐Wallis test was used to compare the distributions of continuous variables by group. All statistical tests were performed at the 5% significance level. All analyses were conducted using SAS, version 9.4 (SAS Institute Inc., Cary, North Carolina).

### 2.1 Ethical considerations

This project (IRB # 16‐703) was reviewed and approved by The Feinstein Institutes for Medical Research Institutional Review Board. The IRB granted a waiver of consent as it considered that it was not practical to obtain informed consent from participants; data was archival. All data was de‐identified. All de‐identified data were provided by the Office of Academic Affairs within the Zucker School of Medicine at Hofstra/Northwell Health.

## 3 RESULTS

Table 1 shows the 2014 to 2015 residency applicants' sociodemographic characteristics (n = 10 541). The study sample included 2707 White, 722 Black, 805 Hispanic, 5006 Asian, and 562 Other Race/Ethnicity applicants. Overall, 50.2% were male, 21.3% were URiM, 7.4% had limited English proficiency, 67.6% attended international medical schools, and 2.4% were Alpha Omega Alpha Honor Medical Society (AOA) members. We observed significant differences in the means of USMLE step 1 score by race and ethnicity groups; non‐Hispanic White applicants had the highest mean (±SD) average, of 225.1 ± 19.9, followed by 222.9 ± 19.1 for Asians, 220 ± 19.3 for Other Race/Ethnicity, 216.3 ± 19.1 for Hispanic, and 212.7 ± 16 for Black applicants (*P* < .01, ANOVA). We also observed significant differences in sex, international medical school attendance, and AOA status by race/ethnicity group. We also observed a significant difference in the distribution of age by race/ethnicity group (Table 1).

**Table 1:** Characteristics of residency applicants by self‐reported race/ethnicity

	Race/ethnicity						*P*‐value^a^
	All	White	Black	Hispanic	Asian	Other	
Mean USMLE step 1 (SD)	222.1 (19.4)	225.1 (19.9)	212.7 (16.0)	216.3 (19.1)	222.9 (19.1)	220.0 (19.3)	<.01
LEP (%)	7.4%	7.9%	9.0%	6.4%	7.2%	7.8%	.24
Male (%)	50.2	52.6	39.4	46.6	49.4	59.7	<.01
Median age	27	27	29	28	27	28	<.01
International medical school (%)	67.6	51.8	61.0	66.3	75.7	74.9	<.01
AOA (%)	2.4	5.8	0.1	1.8	1.3	1.5	<.01

Abbreviations: USMLE = United States Medical Licensing Exam (USMLE), LEP = Limited English Proficiency, AOA = Alpha Omega Alpha Honor Medical Society member. LEP was categorized as Not English native/functionally native or English native/functionally native. International medical school was categorized as Not trained in the US or Trained in the US. AOA was categorized as Yes or No.

a ANOVA was used to compare the mean USMLE Step 1 score by race category. The chi‐square test was used to compare percent LEP, percent male, percent international medical school, and percent AOA by race category. The Kruskal‐Wallis test was used to compare age distribution by race category.

We also examined these characteristics by URiM group (Table 2). We observed a significant difference in the mean (±SD) USMLE step 1 score by URiM group (Non‐URiM = 223.7 ± 19.4 vs URiM = 216.1 ± 18.4, *P* < .01, *t*‐test), and a significant difference in the distributions of age by URIM group (median age [IQR]: non‐URiM = 27 [5] vs URiM = 28 [4], *P* < .01, Wilcoxon Rank Sum test). There were also statistically significant differences in sex and AOA status by URiM group.

**Table 2:** Comparison of residency applicants between URiM vs non‐URiM

	All	Non‐URiM	URiM	*P*‐value†
Mean USMLE step 1 score (SD)	222.1 (19.4)	223.7 (19.4)	216.1 (18.4)	<.01
Median age (IQR)	27 (4)	27 (5)	28 (4)	<.01
LEP (%)	7.4	7.5%	7.7%	.75
International medical school (%)	67.6	67.1	66.8	.78
Male (%)	50.2	50.5	47.7	.02
AOA (%)	2.4	2.9	1.2	<.01

Abbreviations: USMLE = United States Medical Licensing Exam (USMLE), SD = Standard Deviation, IQR = Interquartile Range, LEP = Limited English Proficiency, AOA = Alpha Omega Alpha Honor Medical Society.

^a^The two‐sample *t*‐test was used to compare mean USMLE Step 1 score by URiM status. The Wilcoxon Rank Sum test was used to compare distribution of age by URiM status. The Chi‐Square test was used to compare percent LEP, percent international medical school, percent male, and percent AOA by URiM status. URiM included those in the Black, Hispanic, and Other race/ethnicity categories, and Non‐URM included those in the Asian and White categories.

We then examined whether there were differences in USMLE step 1 score by specialties, and we observed significant differences in the means of USMLE step 1 score by race/ethnicity group within each specialty (Table 3).

**Table 3:** USMLE step 1 score of residency applicants by medical specialties

	Race/ethnicity category					
	White	Black	Hispanic	Asian	Other	*P*‐value†
IM site 1 Mean (SD)	229.1 (20.0) n = 801	215.1 (16.3) n = 178	218.8 (19.2) n = 224	226.4 (18.6) n = 1624	221.8 (18.7) n = 165	<.01
IM site 2 mean (SD)	230.1 (18.9) n = 868	218.1 (15.9) n = 171	222.8 (19.1) n = 248	228.3 (17.8) n = 1831	226.6 (18.1) n = 201	<.01
OB/GYN mean (SD)	218.0 (18.0) n = 325	210.5 (14.6) n = 162	209.3 (15.4) n = 111	215.0 (17.7) n = 229	209.8 (17.5) n = 59	<.01
Pediatrics mean (SD)	222.0 (18.7) n = 427	209.7 (15.8) n = 112	211.4 (18.3) n = 142	217.3 (17.5) n = 615	216.7 (19.8) n = 61	<.01
Psychiatry mean (SD)	212.0 (16.8) n = 286	205.9 (14.2) n = 99	208.2 (16.3) n = 80	208.6 (15.5) n = 707	209.4 (16.9) n = 76	.01

Abbreviations: IM, internal medicine, OBGYN, obstetrics and gynecology.

^a^The ANOVA test was used to compare mean USMLE step 1 score by race/ethnicity category within medical specialty. No post‐hoc analysis was completed to test for multiple comparisons between race/ethnicity categories.

Next, we examined the percentage of applicants with USMLE step 1 scores above the cutoff score in five‐point increments, from 205 to 235 (Table 4). For each cutoff score, mean USMLE step 1 scores significantly differed by racial/ethnic categories. It can be seen that as cutoff scores increase, the proportions of URiMs excluded is significantly higher. For example, 44.4% of Whites meet the 230‐cutoff, compared to 15.7% of Blacks and 25.2% of Hispanics. In comparison, 60.3% of Whites meet the 220‐cutoff compared to 31.4% of Blacks and 40.4% of Hispanics.

**Table 4:** Percentage of applicants with USMLE step 1 score above the cutoff score

Cutoff Score	All	White	Black	Hispanic	Asian	Other	*P*‐value†
205	78.1	81.0	66.3	68.0	80.1	75.1	<.01
210	70.5	74.5	54.9	58.4	72.5	68.2	<.01
215	62.8	68.1	40.9	48.9	65.9	57.8	<.01
220	54.5	60.3	31.4	40.4	57.6	49.5	<.01
225	46.1	53.5	22.7	31.9	48.4	41.6	<.01
230	37.2	44.4	15.7	25.2	38.6	33.1	<.01
235	28.5	35.0	10.1	19.3	29.3	25.3	<.01

^a^The Chi‐Square test was used to compare percentages by race/ethnicity category.

Lastly, for each specialty, the overall mean USMLE step 1 score was calculated. The percentage of URiM and non‐URiM that were above the mean score were calculated. We observed significant differences in the percentage of applicants above the mean between URiM and non‐URiM applicants for IM site 1 (URIM: 34.2%, non‐URiM: 56.3%, *P* < .01), obstetrics (URiM: 34.9%, non‐URIM: 54.7%, *P* < .01), IM site 2 (URiM: 39.2%, non‐URiM 55.3%, *P* < .01), and pediatrics (URIM: 40.0%, non‐URIM: 45.7%, *P* < .01, Chi‐Square test). We observed no statistically significant difference between percentages of applicants within Psychiatry by URiM group (URiM: 40.0%, non‐URiM 45.7%, *P* = .10, Chi‐Square test; Table 5).

**Table 5:** Percentage of residency applicants by URiM who are above the mean USMLE step 1 score

Medical specialties	Mean USMLE step 1 score	All (% above)	Non‐URiM (% above mean)	URiM (% above mean)	*P*‐value†
Internal medicine site 1	225.6	52.1	56.3	34.2	<.01
Obstetrics	214.0	46.9	54.7	34.9	<.01
Internal medicine site 2	227.6	52.1	55.3	39.2	<.01
Pediatrics	217.5	48.5	53.7	32.4	<.01
Psychiatry	209.4	45.2	45.7	40.0	.10

^a^The Chi‐Square test was used to compare percentages by URiM status.

## 4 DISCUSSION

By 2045, the US will be a majority‐minority nation.^28^ Given the changing population and numerous benefits of a diverse workforce, several national medical education organizations as National Academy of Medicine (NAM ‐ formerly Institute of Medicine), Liaison Committee on Medical Education (LCME), and Accreditation Council for Graduate Medical Education (ACGME) have made recommendations for diversification of the physician workforce.^1^, ^2^, ^7^, ^12^, ^29^ One of the four mission areas of the AAMC is diversity and inclusion, and they have published numerous recommendations and resources on ways to achieve diversity. The ACGME's 2019 updated Common Program Requirements^30^ explicitly state that “institution(s) must engage in the (…) recruitment and retention of a diverse and inclusive workforce,” specifically, in the “recruitment and retention of minorities underrepresented in medicine.”^31^ The LCME states that “each medical school must have policies and practices to achieve appropriate diversity among its students, faculty, staff, and other members of its academic community.”^32^, ^33^

Despite lots of recommendations and effort, little progress has been made in creating a truly diverse physician workforce.^1^, ^5^, ^6^, ^14^ The use of cutoffs in USMLE step 1 scores for granting residency interviews may be a contributor to why little progress has been made.^1^, ^11^, ^15^, ^16^ Recently, the USMLE's parent organization (The Federation of State Medical Boards and NBME) and AAMC met with “key stakeholders” to discuss reporting the results of the USMLE step 1 as pass/fail instead of a numeric score.^15^, ^16^, ^17^, ^20^ Reporting the USMLE step 1 scores as pass/fail would meet the primary purpose of the exam, which is licensing, as well as reduce its significant value in resident selection, therefore providing an opportunity to increase diversity of the physician workforce.^14^, ^15^, ^16^

Our study aimed to explore associations between USMLE step 1 scores and URiM status and whether this association persists for different medical specialties, at one of the largest health systems in the US. We purposefully chose USMLE scores for two reasons. First, USMLE scores are a significant part of the selection process in residency programs, and second, there are decades of empirical evidence indicating that standardized testing is problematic for physician ratings and advancement for URiM.^7^, ^11^, ^22^, ^25^, ^34^, ^35^, ^36^, ^37^, ^38^

As hypothesized, our study revealed significant differences in the means of USMLE step 1 scores by race/ethnicity categories and URiM groups. In our cohort, URiM scored lower than their White counterparts on the USMLE step 1 exam, which is consistent with previous studies that showed that White students performed higher on USMLE step 1 than other racial/ethnic minorities^34^, ^39^ This trend was consistent within different applicant specialties. The data from our sample are generalizable to national trends, given it represents applicants from the majority of the US medical schools and applicants from all 50 states. When using the 2014 mean USMLE step 1 score (*X* = 230) for matched applicants, 84.3% of Black applicants and 74.8% of Hispanic applicants would be eliminated from the pool.^40^

The use of USMLE step 1 cutoff scores as a recruitment and selection criteria contributes to the “leakiness of the pipeline,” that is, the departure of students, particularly minorities, from a medical career path.^12^, ^13^ Though multifactorial, the “leaky pipeline” posits that minority students who have an interest in science, technology, engineering, and mathematics (STEM) careers change their minds when applying to college or leave the “pipeline” after graduating with a STEM degree due to negative experiences such as microaggressions and discrimination.^22^, ^41^, ^42^, ^43^, ^44^ Lower scores on standardized tests such as USMLE may also be a contributing factor to the leaky pipeline, with a consequence of that leakage being lack of representation of physicians from racial/ethnic minority backgrounds, who speak languages other than English and are representative of the U.S. patient population they serve.^12^, ^13^, ^14^, ^42^, ^45^

This study has several limitations. Although our applicants are diverse and from all parts of the US, it is important to note that we only examined individuals applying to one (albeit large) institution. The applicant pool might be different for other institutions, and study findings may not be generalizable. In addition, our data included applicants in the ERAS for 2014 to 2015 and may not represent the current demographics of applicants. Similar to widely used practices,^14^, ^26^, ^29^, ^45^ each of the specialties at our institution represented in this cohort, in this study year, did use, in one way or another, a USMLE cutoff that influenced which applications were reviewed and/or which applicants were offered interviews. Future research should revisit these analyses across institutions and years as well as standardized tests (eg, COMLEX), to provide a more comprehensive view of diversity in medicine.

## 5 PRACTICAL IMPLICATIONS

To better understand physician diversity, academic researchers and program directors should focus on attaining a more in‐depth perspective of the recruitment and selection process experienced by URiM.^14^, ^15^, ^16^, ^45^ During the recruitment and selection process, structural barriers, microaggressions, biases, and cutoff scores from standardized test scores such as USMLE step 1 scores might hinder the acceptance and progress of URiM medical students throughout their medical career.^2^, ^12^, ^13^ According to the leaky pipeline theory, a consequence of the leakage of the diverse talent pool is the underrepresentation of minorities in the medical field.^12^ For program directors, this research helps to take a more closer look at the recruitment and selection process, which would help increase diversity in the physician workforce.^1^, ^7^, ^11^, ^15^, ^16^

As reported earlier, there is growing evidence stating that minority patients report high levels of better communication, satisfaction, and adherence to medication when they are treated by physicians who are similar to them.^1^, ^2^, ^5^, ^7^ Such gains to reducing health disparities are of growing importance to payers, providers, and health plans.^1^, ^2^, ^29^ Medical schools can increase the diversity of the physician workforce to treat a racially/ethnically diverse population by redoubling efforts to recruit URiM. However, in an effort to do so, program directors and medical schools may need to change the way they evaluate medical school applicants.^1^

The recent announcement to report USMLE step 1 scores from three‐digit numeric scores to pass/fail is a vital step.^17^, ^20^ Maintaining the status quo of numeric scores works against the initiatives to increase physician diversity. As shown in our own and previous research, compared to Asian and White students, URiM score lower on step 1.^11^, ^26^ Very often, step 1 scores are used as an alternative to one's work ethic, time management skills, and determination, despite there being a lack of empirical evidence to explain systematic differences.^15^ Additionally, recent empirical evidence shows that students with higher USMLE step 1 scores often self‐select into more competitive specialties^16^; as a result, diverse candidates are more than likely being screened out.^15^, ^46^ Medical schools which have thus far implemented a pass/fail curriculum reporting of results have reported an increase in student well‐being without any effect on academic achievement.^14^, ^47^

In an attempt to diversify the physician workforce, program directors and residency programs can, instead, engage in a more holistic application review. With a holistic application process, program directors can create a rubric for ranking participants which tailors more specifically to the mission and values of the program and institution instead of being so highly dependent on pure numerical scores.

## 6 CONCLUSION

This study demonstrated that using a USMLE step 1 score as an initial filter for applicant recruitment and selection could jeopardize the benefits of a diverse residency program. As indicated by our results, using a fixed cutoff score will significantly reduce the percentage of URiM who are selected into residency programs. Including URiM in the workforce/learning environment has been shown to improve learning for all,^38^ assist with addressing the shortage of physicians in underserved areas,^7^ and increase patient satisfaction and cultural competence. Given our results and other empirically derived data about the unintended consequences of the USMLE step 1 score, residency programs should carefully consider how the USMLE step 1 score should be used in the application process. USMLE step 1 was “not designed to, nor does it predict, the success of a physician.”^48^ When we use the USMLE score or any other one‐dimensional score as a cutoff, we are losing out on qualified URiM who will help both reduce health disparities and improve diversity across medical institutions. Most importantly, we limit the opportunity to evaluate applicants on other criteria which, in the end, may represent more important characteristics of an exceptional healer.

## CONFLICT OF INTEREST

The authors declare that there is no conflict of interest regarding the publication of this paper.

## TRANSPARENCY STATEMENT

Myia Williams affirms that this manuscript is an honest, accurate, and transparent account of the study being reported; that no important aspects of the study have been omitted; and that any discrepancies from the study as planned (and, if relevant, registered) have been explained.

## AUTHOR CONTRIBUTIONS

Conceptualization: Myia Williams, Renee Pekmezaris, Johanna Martinez

Data Curation: Myia Williams, Eun Ji Kim, Karalyn Pappas, Johanna Martinez

Formal Analysis: Myia Williams, Karalyn Pappas

Investigation: Myia Williams, Eun Ji Kim, Renee Pekmezaris, Johanna Martinez

Methodology: Myia Williams, Omolara Uwemedimo, Renee Pekmezaris, Johanna Martinez

Project Administration: Myia Williams, Johanna Martinez

Resources: Omolara Uwemedimo, Johanna Martinez

Supervision: Renee Pekmezaris, Johanna Martinez

Validation: Myia Williams, Eun Ji Kim, Karalyn Pappas, Omolara Uwemedimo, Lyndonna Marrast, Renee Pekmezaris, Johanna Martinez

Visualization: Myia Williams, Eun Ji Kim, Karalyn Pappas

Writing — Original Draft Preparation: Myia Williams, Eun Ji Kim, Karalyn Pappas, Omolara Uwemedimo, Lyndonna Marrast, Renee Pekmezaris, Johanna Martinez

Writing — Review & Editing: Myia Williams, Eun Ji Kim, Karalyn Pappas, Omolara Uwemedimo, Lyndonna Marrast, Renee Pekmezaris, Johanna Martinez

All authors have read and approved the final version of the manuscript. Myia Williams had full access to all of the data in this study and takes complete responsibility for the integrity of the data and the accuracy of the data analysis.

7

## Data Availability

Research data are not shared. Restrictions apply to the availability of these data, which were used under the jurisdiction of one of the co‐authors of this study. The data are not publicly available due to privacy or ethical restrictions.
